# GCN2-Mediated eIF2α Phosphorylation Is Required for Central Nervous System Remyelination

**DOI:** 10.3390/ijms26041626

**Published:** 2025-02-14

**Authors:** Paulina Falcón, Álvaro Brito, Marcela Escandón, Juan Francisco Roa, Nicolas W. Martínez, Ariel Tapia-Godoy, Pamela Farfán, Soledad Matus

**Affiliations:** 1 Fundación Ciencia & Vida, Avenida del Valle 725, Huechuraba, Santiago 8580704, Chile; paulina.falcon.u@gmail.com (P.F.); alvarobrito43@gmail.com (Á.B.); mescandon@cienciavida.org (M.E.); juan.roap@utem.cl (J.F.R.); nmartinez@cienciavida.org (N.W.M.); atapia@cienciavida.org (A.T.-G.); pfarfan@cienciavida.org (P.F.); 2 Ph.D. “Program in Cell Biology and Biomedicine”, Facultad de Medicina y Ciencia, Universidad San Sebastián, Santiago 7510157, Chile; 3 Centro Ciencia & Vida, Fundación Ciencia & Vida, Santiago 8580704, Chile; 4 Facultad de Medicina y Ciencia, Universidad San Sebastián, Santiago 7510157, Chile

**Keywords:** GCN2, myelin, ISR, eIF2α, cuprizone, remyelination

## Abstract

Under conditions of amino acid deficiency, mammalian cells activate a nutrient-sensing kinase known as general control nonderepressible 2 (GCN2). The activation of GCN2 results in the phosphorylation of the alpha subunit of the eukaryotic initiation factor 2 (eIF2α), which can be phosphorylated by three other three integrated stress response (ISR) kinases, reducing overall protein synthesis. GCN2 activation also promotes the translation of specific mRNAs, some of which encode transcription factors that enhance the transcription of genes involved in the synthesis, transport, and metabolism of amino acids to restore cellular homeostasis. The phosphorylation of eIF2α has been shown to protect oligodendrocytes, the cells responsible for producing myelin in the central nervous system during remyelination. Here, we explore the potential role of the kinase GCN2 in the myelination process. We challenged mice deficient in the GCN2-encoding gene with a pharmacological demyelinating stimulus (cuprizone) and evaluated the recovery of myelin as well as ISR activation through the levels of eIF2α phosphorylation. Our findings indicate that GCN2 controls the establishment of myelin by fine-tuning its abundance and morphology in the central nervous system. We also found that GCN2 is essential for remyelination. Surprisingly, we discovered that GCN2 is necessary to maintain eIF2α levels during remyelination.

## 1. Introduction

Mammals have developed a complex network for integrating different types of stresses, the integrated stress response (ISR), where four kinases sense a wide variety of stress conditions converging into a unique event, which is the phosphorylation of the alpha subunit of the eukaryotic translation initiation factor 2 (eIF2α) by four ISR sensors/kinases: general amino acid control nonderepressible 2 (GCN2), heme-regulated inhibitor (HRI), double-stranded RNA-dependent protein kinase (PKR), and PKR-like ER kinase (PERK) [1]. This phosphorylation event, in turn, induces global protein synthesis arrest, which conserves cellular resources and activates a protective response by jointly promoting the translation of certain mRNAs, such as the one encoding the activating transcription factor 4 (ATF4), which will drive the expression of target genes further on [2,3]. The signaling of the ISR is negatively regulated by the action of GADD34 (PPP1R15A), a stress-induced regulatory subunit of the protein phosphatase 1 (PP1) that dephosphorylates eIF2α [4]. Nutritional stress activates the ISR, particularly amino acid deprivation, which activates the GCN2 kinase [5,6]. GCN2 activation leads to the phosphorylation of eIF2α [7] and activates a program of genes involved in amino acid metabolism, redox maintenance, and autophagy, among others, to manage metabolic stress [8].

How GCN2 contributes to central nervous system (CNS) architecture, function, and dynamic maintenance is a topic that has been explored in mice lacking the GCN2 gene (GCN2^−/−^). These mice do not exhibit any noticeable phenotype under normal conditions, but they show significant changes when stressed [9]. For instance, GCN2^−/−^ mice exhibit impairments in long-term spatial memory following intense training, highlighting the importance of GCN2 in synaptic plasticity [10]. Additionally, other studies have identified GCN2 as crucial for signaling in response to amino acid deficiency in the brain, [11] where it helps omnivores to detect amino acid imbalances, triggering aversive responses to nutrient-deficient diets [12,13]. One interesting piece of evidence was described by Tracy G. Anthony’s group. They observed a leukodystrophy phenotype (white matter defects) in GCN2^−/−^ mice with a genetic deficiency in branched-chain amino acids catabolism. This suggests that nutritional stress sensing might contribute to CNS phenotypes supported by the oligodendrocytes [14]. However, whether GCN2 is involved in the function of the oligodendrocytes is still unknown. 

Oligodendrocytes are the myelinating cells in the CNS of vertebrates [15]. Each oligodendrocyte can myelinate up to 50 axonal segments, depending on the region of the CNS [16], by extending their processes over large areas, wrapping axons, and, finally, generating myelin sheaths (reviewed in [16,17]). This myelinating process involves the synthesis of a myriad of proteins and lipids, which demands a secretory [18] and energy capacity [19] in the oligodendrocytes. Initially, the major function of the oligodendrocytes was thought to be the generation of electrical insulators. However, their essential role in providing metabolic support to neurons has been established (reviewed in [20]). For example, oligodendrocytes supply energy to neurons through monocarboxylate transporters and assist in maintaining axonal integrity [21,22]. Despite the oligodendrocytes’ role as metabolic suppliers and the proteostatic changes they experience during myelination, a functional relationship between metabolic stress and oligodendrocyte function or dysfunction remains unclear. Most evidence linking GCN2 and oligodendrocyte functions comes from studies using mouse models of multiple sclerosis (MS). MS is an inflammatory demyelinating disease where the death of the oligodendrocytes contributes to its pathogenesis [23]. Research indicates that GCN2 contributes to the development of the remission phase in experimental autoimmune encephalomyelitis (EAE), one of the MS models [24]. After EAE induction, through immunization with a peptide from the myelin oligodendrocyte glycoprotein (MOG) [25], mice lacking GCN2 exhibit alterations not only in immune processes but also in the remyelination process [24]. Consequently, these GCN2-deficient mice do not fulfill the remission phase following EAE induction [24]. In the same inflammatory context of EAE, a study demonstrated that the eIF2α phosphorylated levels are increased in neurons during the peak of inflammation [26]. This phosphorylation event was critical since its inhibition was linked with remyelination impairment and defects in remission accomplishment [26]. These data highlight the significance of ISR activation (i.e., eIF2α phosphorylation) in the myelination process and oligodendrocyte biology. Another study highlighted the importance of the phosphorylation of eIF2α during remyelination [27]. Chen et al. used Sephin1, a small molecule that inhibits the dephosphorylation of eIF2α [28], in the MOG-induced EAE mouse model [27]. Sephin1 delayed the onset of clinical symptoms. This delay was associated with a prolonged ISR activation, reduced loss of oligodendrocytes and axons, and decreased T cell presence in the CNS [27]. In other studies, the researchers investigated the effects of enhancing eIF2α phosphorylation, either genetically using a mouse deficient in the eIF2α phosphatase encoding gene (GADD34-KO mouse) [29] or pharmacologically (using Sephin1) [29,30] on remyelination. They modeled MS in mice by combining the ectopic IFN-γ expression in the CNS to emulate the inflammatory condition and the cuprizone-induced demyelinating model, a drug that promotes the loss of oligodendrocytes [31,32]. They showed that promoting eIF2α phosphorylation protects remyelinating oligodendrocytes that repopulate after cuprizone treatment and consequently promote remyelination. However, whether the eIF2α kinase GCN2 participates in this protective phenotype has not been explored. Here, we studied the role of GCN2 on basal myelin status as well in the remyelination process. We used the cuprizone-induced pharmacological demyelinating assay, a well-established pharmacological model [31,32], to assess the remyelination capabilities of GCN2-deficient mice. While the exact mechanism of cuprizone’s action remains unclear, it is known to cause cellular damage and subsequent cell death, particularly in oligodendrocytes. Additionally, cuprizone acts as a copper chelator, further contributing to its toxic effects [32]. First, our results indicate that GCN2 contributes to regulating the abundance and size of essential myelin components in basal conditions. Second, we found that mice lacking GCN2 are more sensitive to the cuprizone treatment and that demyelination and remyelination were differentially regulated. Third, we found that the recovery of eIF2α levels in WT mice during remyelination withdrawal is not observed in GCN2 deficiency, revealing that GCN2 controls the expression and abundance of the stress response integrator eIF2α during remyelination.

## 2. Results

### 2.1. GCN2 Contributes to the Myelin Phenotype in the CNS

**Figure 1:**
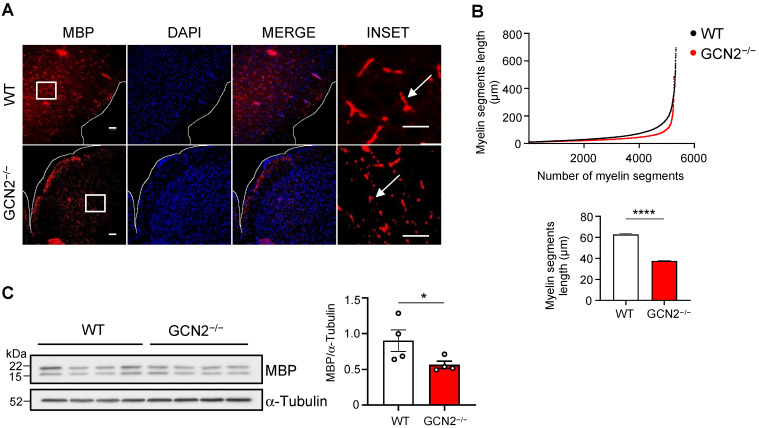
GCN2 deficiency impacts the length of myelinated segments and myelin levels. Sections (30 μm) of the brain derived from two-month-old male WT (n = 3) and genetically deficient GCN2 (GCN2^−/−^, n = 3) mice were obtained and processed for immunofluorescence to visualize myelin segments. (**A**) Representative images showing the myelin basic protein (MBP) (red) staining used as a myelin marker, the nuclei DAPI (blue), and the composed image (merge). An augmented section (white box) of the images are shown at the right (Zoom). Arrows on the zoom image show the type of MBP-positive objects quantified. (**B**) Images obtained in A were quantified using the Image J software (1.53t version). The plot shows the number and length of myelin segments (upper panel) and the average length (lower panel) of myelin segments found in WT and GCN2^−/−^ mice. (**C**) Protein extracts were prepared from brains derived from two-month-old WT (n = 4) and GCN2^−/−^ (n = 4) mice. MBP levels were analyzed by Western blot using α-Tubulin as a loading control (left). Each lane represents an individual (30 μg/lane). Densitometry analysis of the blot was performed by ImageJ to quantify the intensities of the two MBP bands and then normalized to α-Tubulin (right) levels. Scale bar: 100 μm. **** *p* < 0.00001, * *p* < 0.05. *t*-test analysis. Error bars represent the mean ± SEM. The whole Western blot membrane is shown in Supplementary Figure S1. The MBP quantification strategy is described in the “Materials and Methods” section.

First, we assessed the role of GCN2 in one of the main oligodendrocyte functions, myelination, in basal conditions. To this goal, we determined levels of myelin and length in animals lacking the gene encoding for GCN2 (GCN2^−/−^) and compared them with those from WT mice. We analyzed the number and length of the cortical myelinated segments using immunodetection of the myelin basic protein (MBP), a ubiquitous myelin protein (Figure 1A). In the sections analyzed, we found a similar number of myelin segments (5332 segments in WT, 5263 segments in GCN2^−/−^). However, our analysis showed a significant reduction in the mean length of the myelinated segments in the cortex of GCN2^−/−^ mice compared to WT mice. An average length of 62 μm was found in the WT cortex, decreasing to an average of 37 μm in the GCN2^−/−^ mice (Figure 1B). Furthermore, when we addressed the levels of myelin protein in the cortex, we found a decrease of 37.3% in MBP levels in GCN2^−/−^ mice (Figure 1C). These findings strongly suggest that GCN2 contributes to the myelin phenotype in adult mammals by fine-tuning its abundance and the length of myelin segments in the CNS.

### 2.2. The Remyelination Process Is Impaired in GCN2-Deficient Mice

**Figure 2:**
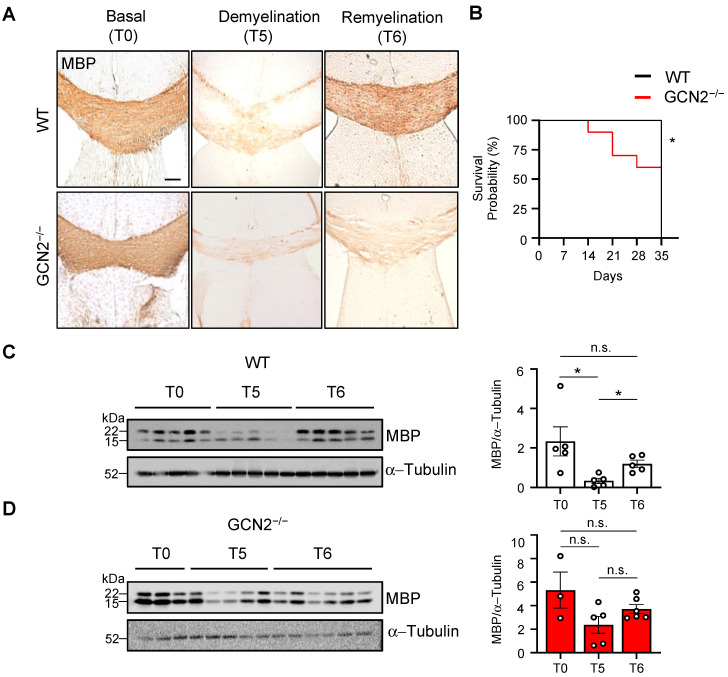
GCN2 is necessary for CNS remyelination. Two-month-old WT (males) and GCN2 knockout (GCN2^−/−^) mice (males) were treated with 0.2% cuprizone mixed into their food for 5 weeks (T5). After this treatment period, cuprizone was removed from their diet, allowing remyelination to occur for 1 week (T6). (**A**) Representative images of immunohistochemical staining of the corpus callosum of WT and GCN2^−/−^ mice at basal (T0), T5, and T6. An antibody specific to myelin basic protein (MBP) was used. Scale bar 100 μm. n = 3 for all time points (two-month-old animals) (**B**) Kaplan–Meier survival curve of WT (n = 10) and GCN2^−/−^ (n = 10) until T5. * *p* < 0.05. Log-rank Mantel–Cox test. (**C**) Myelin levels were assessed by detecting MBP through Western blot analysis in protein extracts from the cerebral cortex in WT untreated mice (T0) n = 5, after 5 weeks of treatment (T5) n = 5, and after 1 week of recovery following cuprizone treatment (T6) n = 5. Two-month-old WT and GCN2^−/−^ male animals for all conditions. On the right, a densitometric analysis of the blots was performed using ImageJ to quantify the two MBP band intensities, which were normalized to α-Tubulin levels. n.s.: non-significative differences. * *p* < 0.05. Mann–Whitney test. Error bars represent the mean ± SEM. * *p* < 0.01 Figure 1. The whole Western blot membranes are shown in Supplementary Figure S2. (**D**) In an independent experiment, myelin levels were assessed by detecting MBP through Western blot analysis in protein extracts from the cerebral cortex of two-month-old GCN2^−/−^ mice. MBP detection in protein extracts from GCN2^−/−^ mice at T0 (n = 3), T5 (n = 5), and T6 (n = 6). Each lane represents an individual sample, with α-Tubulin as a loading control. A total of 30 µg of protein was loaded per lane. On the right, the densitometric analysis of the Western blots was performed as in (**C**). n.s.: non-significative differences. * *p* < 0.05. Mann–Whitney test. Error bars represent the mean ± SEM. * *p* < 0.01 Figure 1. The whole Western blot membranes are shown in Supplementary Figure S2.

We investigated the role of GCN2 in the remyelination process by challenging GCN2^−/−^ mice and their WT littermates to revert demyelination caused by cuprizone intoxication. This treatment is a well-established model for studying remyelination, as previously described ([33,34,35] reviewed in [36]). Cuprizone causes oligodendrocyte damage and consequent death [31], leading to reversible demyelination of the CNS when administrated orally over a period of 5 weeks ([37], reviewed in [36]). Both WT and GCN2 genetically deficient mice were given cuprizone (0.2%) mixed with the standard powdered chow for 5 weeks to induce demyelination. We evaluated myelination status through MBP immunochemistry in the corpus callosum, a region particularly sensitive to cuprizone treatment [37]. Both mice showed a histological staining pattern compatible with a complete myelination (T0, Figure 2A). After 5 weeks of treatment, a decrease in myelin staining levels was observed in WT and GCN2-deficient animals (T5, Figure 2A), indicating that in the corpus callosum, the drug promoted demyelination. We also observed changes in corpus callosum size. Important, while WT mice did not exhibit any notable health issues during the 5-week cuprizone challenge, a considerable proportion of GCN2-deficient mice displayed clear signs of general distress detected by severe kyphosis, eye closure, and ruffled fur. Given these observations, we assigned a score to these three parameters for a semi-quantitative analysis. GCN2-deficient animals scored higher for all parameters (Supplementary Figure S3), indicating a systemic alteration. Moreover, some GCN2-deficient animals succumbed to the cuprizone treatment. Specifically, 40% of the GCN2-deficient group did not survive the demyelinating challenge (Figure 2B). The remainder individuals managed to withstand the drug’s effects with evident affectation on their motor performance. Importantly, all WT animals that underwent treatment remained in good condition, without motor affectation, and none died during the study. After one week of cuprizone removal (T6), the MBP recovery observed in the corpus callosum of WT mice was completely absent in the GCN2-deficient mouse (T6, Figure 2A), indicating that GCN2 is necessary for remyelination after the pharmacological demyelinating treatment. 

To further evaluate the demyelination and remyelination process, we examined the changes in myelin in mice at three stages of the treatment: without intervention (T0), after five weeks of cuprizone treatment (T5), and at the end of the sixth week when full remyelination typically occurs (T6) in the cortex. The myelination status at each point was measured by assessing MBP levels by Western blot assay. First, we analyzed changes in MBP levels in WT mice. We observed a significant decrease in MBP levels after exposure to cuprizone (T0–T5), but these levels recovered upon cessation of the treatment, indicating successful remyelination in the cortex (T5–T6, Figure 2C). Then, we evaluated the demyelination and remyelination process in GCN2-deficient mice (Figure 2D), similar to the analysis for the WT mice but in an independent experiment. Unexpectedly, GCN2-deficient mice did not show statistical differences in MBP levels after 5 weeks of cuprizone treatment compared to basal (T0–T5), indicating less sensibility to demyelination, possibly due to inherently lower myelin levels in the cortex, as previously detected (Figure 1C). After the sixth week, there was no increase in MBP levels compared to those observed after 5 weeks of cuprizone, showing that GCN2-deficient mice do not respond to drug removal. These findings show that the cortical oligodendrocytes of GCN2-deficient mice myelinate and respond to cytotoxic challenges in a different manner than in WT mice and suggest that GCN2 participates in the susceptibility to cuprizone treatment, demyelinating in corpus callosum but insensitive in the cortex. Of note, the animals lacking GCN2, which were able to withstand the cuprizone treatment, exhibited high variability in their MBP content after the cuprizone challenge (T5) and during the subsequent recovery period (T6) (Figure 2C). Thus, we not only observed differences in the GCN2-deficient animals (susceptible and non-susceptible individuals to cuprizone treatment) but also in those animals that managed the treatment. When we compared the MBP fold change between WT and GCN2-deficient animals, no differences were observed (Supplementary Figure S2C). Together, these results show that GCN2 participates in the control of MBP levels differentially in the brain, and GCN2 is crucial for normal remyelination in the corpus callosum. In the cortex, GCN2 provides resistance to cuprizone-induced demyelination.

### 2.3. GCN2 Is Necessary for eIF2α Recovery and Phosphorylation During Remyelination

**Figure 3:**
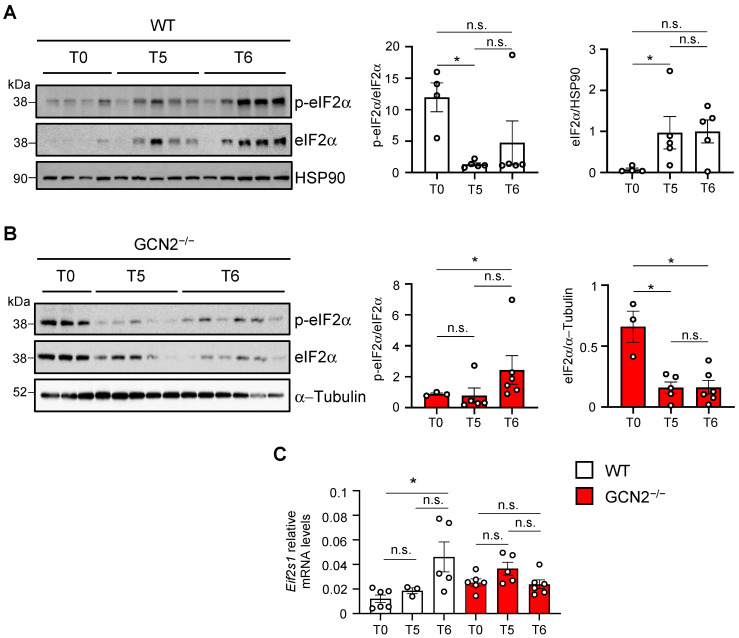
eIF2α phosphorylation levels are impaired during the demyelination/remyelination process in the GCN2 deficient mice. Two-month-old WT and GCN2^−/−^ male mice were treated with cuprizone, and the levels of the α subunit of the eukaryotic translation initiation factor 2 (eIF2α) and its phosphorylated form (p-eIF2α) were analyzed in protein extracts from the cerebral cortex using Western blot. (**A**) p-eIF2α and eIF2α levels on extracts of WT mice untreated (T0, n = 4), after 5 weeks of treatment with cuprizone (T5, n = 5), and at the sixth week of regular chow after 5 weeks of treatment (T6, n = 5). (**B**) Analysis of p-eIF2α and eIF2α levels in GCN2^−/−^ mice untreated (T0, n = 3), after 5 weeks of treatment (T5, n = 5), and at the sixth week of regular chow after 5 weeks of treatment (T6, n = 6). Each lane represents one animal (30 µg/lane). Plots on the right on (**A**) and (**B**) show the densitometry analysis of the blots performed by ImageJ to quantify p-eIF2α band intensities normalized to eIF2α previously normalized with HSP90 for (**A**) and α-Tubulin for (**B**). (**C**) *Eif2s1* transcript levels were evaluated on total mRNA obtained from the cerebral cortex of WT mice untreated (T0, n = 6), after 5 weeks of treatment with cuprizone (T5, n = 3), and at the sixth week of regular chow after 5 weeks of treatment (T6, n = 5) and from GCN2^−/−^ mice untreated (T0, n = 6), after 5 weeks of treatment with cuprizone (T5, n = 5), and at the sixth week of regular chow after 5 weeks of treatment (T6, n = 6). Male two-month-old animals were used in all conditions. The plot shows *Eif2s1* levels normalized against *Actin* transcript levels. Mann–Whitney test. n.s.: non-significative differences. * *p* < 0.05. Error bars represent the mean ± SEM. The whole Western blot membranes are shown in Supplementary Figure S4.

After observing differences in response to cuprizone demyelinating treatment in the CNS between WT and GCN2-deficient mice, we examined the signaling pathway downstream of GCN2 activation during both demyelination and remyelination in the cortex in the WT mice and then analyzed the GCN2-deficient mice. Specifically, we assessed the phosphorylation status of eIF2α (p-eIF2α), the primary downstream effector of the GCN2 signaling pathway, in both WT and GCN2 deficient mice. First, when analyzing eIF2α phosphorylation in the WT mice, the Western blot showed an increase in its levels after 5 weeks of cuprizone (T5) and during the subsequent drug withdrawal (T6) (Figure 3A, left panel). However, eIF2α phosphorylation quantification against total eIF2α (Figure 3A, right panel) showed a decrease in p-eIF2α levels after the cuprizone challenge, and those levels were maintained after the removal of the drug. This result is probably due to the changes observed in eIF2α total levels, which were increased after the 5 weeks of treatment and kept their high levels after taking out the cuprizone (Figure 3A, right panel). Once we defined the dynamic of eIF2α phosphorylation in the WT mice, we analyzed the signaling pathway in mice lacking GCN2. When we analyzed eIF2α phosphorylation dynamics in cortex tissue derived from the GCN2-deficient mice under cuprizone-induced demyelination and remyelination, the Western blot showed the opposite result. We observed decreased phosphorylated and total eIF2α levels over the treatment (Figure 3B, left panel). However, the quantification of the intensity of the phosphorylated bands against total eIF2α (Figure 3B, right panel) showed that eIF2α phosphorylation levels increased in the sixth week. Unlike what was observed in the WT mice, in which there is a substantial increase in total eIF2α levels in mice lacking GCN2, there was a drastic reduction in eIF2α levels during the cuprizone challenge. After cuprizone withdrawal, GCN2-deficient mice cannot recover eIF2α levels. Thus, after a demyelinating stimulus, GCN2-lacking mice lost the capacity to recover the signaling integrator of the stress pathway and, consequently, to promote the GCN2-dependent response that allows remyelination.

Given the loss of total eIF2α protein at the end of the cuprizone treatment and the sixth week, we examined the transcript levels of eIF2α (*Eif2s1*) during the treatment in both genotypes (Figure 3C). WT mice exhibited a substantial increase in *Eif2s1* transcript levels in the sixth week, whereas GCN2-deficient mice showed no changes over time in *Eif2s1* levels (Figure 3C). When *Eif2s1* levels were compared at each time point between the two genotypes, we found that GCN2-deficient mice showed increased levels on basal conditions and after the cuprizone treatment, without changes after drug withdrawal (Supplementary Figure S5), suggesting a compensation mechanism in conditions of eIF2α decreased levels. Thus, we concluded that the kinase GCN2 is necessary for the normal eIF2α protein recovery and phosphorylation during remyelination processes. These results also suggest that the kinase GCN2 regulates the expression of eIF2α after a demyelinating stimulus.

**Figure 4:**
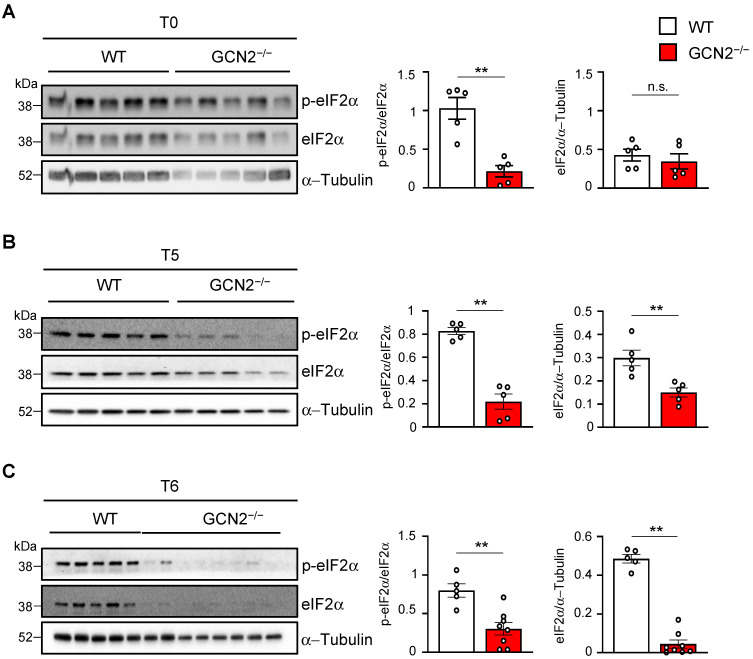
eIF2α levels and phosphorylation are impaired during the remyelination process in the GCN2 deficient mouse. The levels of the α subunit of the eukaryotic translation initiation factor 2 (eIF2α) and its phosphorylated form (p-eIF2α) between WT (males) and GCN2 deficient (males) mice (GCN2^−/−^) were analyzed using Western blot at basal conditions (T0), after treatment with cuprizone for five weeks (T5), and at the sixth week of regular chow after 5 weeks of treatment (T6). Two-month-old animals for all conditions were used. (**A**) Western blot of protein extracts obtained from the cerebral cortex of non-treated (T0) WT (n = 5) and GCN2^−/−^ (n = 5) mice. (**B**) Western blot of protein extracts obtained from the cerebral cortex of WT (n = 5) and GCN2^−/−^ (n = 5) mice from animals treated with cuprizone for five weeks (T5). (**C**) Western blot of protein extracts obtained from the cerebral cortex of WT (n = 5) and GCN2^−/−^ (n = 8) mice subjected to demyelination by cuprizone for five weeks plus one week without treatment (T6). For A, B, and C, the plots on the right show the quantification of the intensity of the bands obtained on the Western blot. For p-eIF2α quantification, the intensity was normalized against the total levels of eIF2α. The intensity of the eIF2α bands was normalized against α-Tubulin. Mann–Whitney test; ** *p* < 0.01; n.s. non-significative differences. Error bars represent the mean ± SEM. Each lane represents one animal (40 µg/lane). The whole Western blot membranes are shown in Supplementary Figure S6. For all membranes, p-eIF2α was first detected, and then the membrane was stripped and incubated with the eIF2α antibody. The last step was loading control detection (α-Tubulin or HSP90).

The different dynamics observed in eIF2α levels and phosphorylation between WT and GCN2-deficient mice treated with cuprizone prompted us to compare total and phosphorylated levels across both genotypes at each point in the cortex on the same membrane. Under basal conditions, lower levels of eIF2α phosphorylation were found in GCN2-deficient mice compared to WT mice (Figure 4A). We did not observe any differences in total eIF2α levels between the two groups. Since eIF2α levels during the demyelination/remyelination process exhibit opposite dynamics between WT and GCN2-deficient mice, the apparently high levels of basal eIF2α detected in Figure 3B compared to levels in WT mice (Figure 3B) are likely due to exposure time. When comparing eIF2α activation (phosphorylation) after acute demyelination (T5) between the genotypes, the phosphorylated fraction of eIF2α significantly decreased in the GCN2-deficient mice compared to WT mice (Figure 4B). Notably, during the demyelination phase (T5), total eIF2α levels also dropped in the GCN2-deficient mouse compared to the WT condition (Figure 4B). Furthermore, when examining eIF2α activation at the remyelination period (T6) between both genotypes, both the phosphorylated fraction and the total protein were markedly decreased in GCN2-deficient mice (Figure 4C). These results show that GCN2 is activating the ISR during both demyelination and remyelination. In the context of demyelination and myelination in the cortex, GCN2 also serves as a proteostatic controller of eIF2α.

### 2.4. Oligodendrocyte Differentiation Markers Are Altered During the Demyelination/Remyelination Process in the GCN2 Deficient Mouse

**Figure 5:**
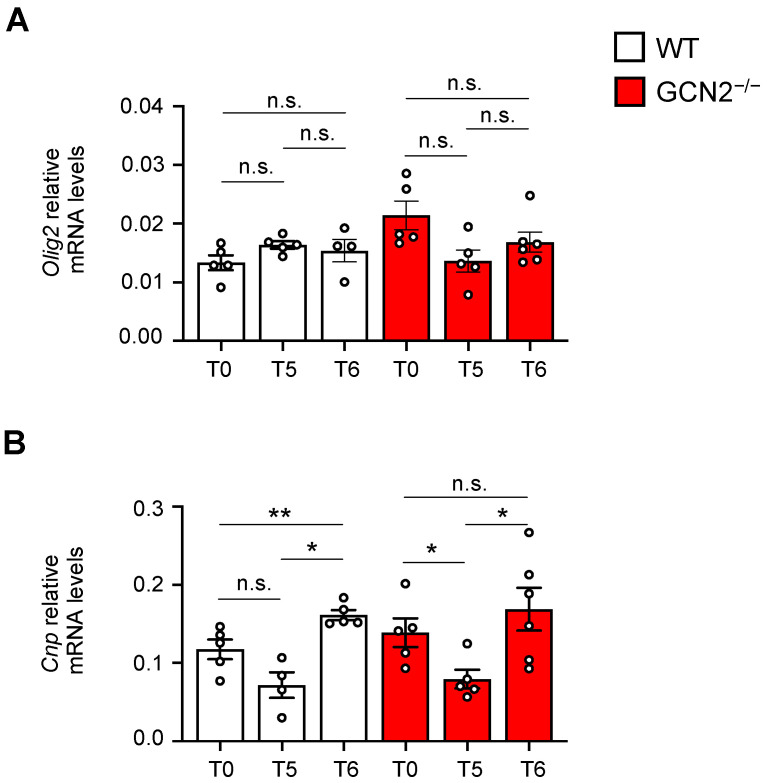
Oligodendrocyte differentiation markers are diminished upon demyelination/remyelination in the *GCN2* deficient mouse. Two-month-old male WT and GCN2^−/−^ mice were treated with cuprizone, and the levels of differentiation markers were analyzed by qPCR in basal conditions (T0), after five weeks of cuprizone treatment (T5), and at the sixth week of regular chow after 5 weeks of treatment (T6). (**A**) Transcript levels of *Olig2* and (**B**) *Cnp* for WT untreated mice (*Olig2* T0, n = 5; *Cnp* T0, n = 5), after 5 weeks of treatment (*Olig2* T5, n = 4; *Cnp* T5, n = 4), and after 1 week of recovery after the CPZ treatment (*Olig2* T6, n = 5; *Cnp* T6, n = 5). For GCN2^−/−^ untreated mice (*Olig2* T0, n = 5; *Cnp* T0, n = 5), after 5 weeks of treatment (*Olig2* T5, n = 5; *Cnp* T5, n = 4), and after 1 week of recovery after the CPZ treatment (*Olig2* T6, n = 6; *Cnp* T6, n = 6). Levels of the transcript analyzed were normalized against *Actin* transcript levels. Mann–Whitney test; * *p* < 0.05; ** *p* < 0.01; n.s. non-significative differences. Error bars represent the mean ± SEM.

We next evaluated the impact of the loss of the GCN2 coding gene on the oligodendrocytes, the cells responsible for producing myelin. To do this, we assessed markers associated with oligodendrocyte function. Specifically, we measured the transcript levels of *Olig2*, a transcription factor that drives the maturation of oligodendrocyte progenitor cells [38,39], as well as *Cnp*, which is a marker for oligodendrocyte maturation before myelinating [40]. We analyzed the dynamics of these transcripts under basal conditions after five weeks of treatment and during the sixth week of recovery in both WT and GCN2-deficient mice. For *Olig2*, we found that both WT and GCN2-deficient mice exhibited similar behaviors. The transcript levels of Olig2 remained unchanged throughout the demyelination and remyelination processes (Figure 5A). For *Cnp*, transcript levels did not change after 5 weeks of treatment in WT mice. However, in the sixth week, *Cnp* levels increased compared to the basal, consistent with a remyelination phenotype [41]. In contrast, GCN2-deficient mice displayed a different pattern. After 5 weeks of treatment, *Cnp* levels were decreased compared to untreated mice (T0) (Figure 5A). Although they increased in the sixth week, they did not reach the levels observed in WT mice (Figure 5A), indicating a reduced capacity of GCN2-deficient mice to properly generate myelin. We also compared the transcript levels of *Olig2* and *Cnp* between WT and GCN2-deficient mice at each time point. In basal conditions (T0), we found increased levels of the precursor proliferation marker *Olig2* in adult GCN2-deficient mice, but no differences were observed during the demyelination and remyelination phases (T5 and T6) (Supplementary Figure S7). The increased levels of *Olig2* could be reflecting a compensation phenotype due to low myelin levels in basal conditions [42]. For the *Cnp* transcript, we noted no differences in GCN2-deficient mice, neither under basal, demyelination, or remyelination conditions, compared to WT mice (Supplementary Figure S7). Together, these results suggest that GCN2 could be involved in oligodendrocyte myelination function and also in oligodendrocyte early stages of differentiation after stress.

## 3. Discussion

While earlier studies have explored the function of GCN2 in the brain (reviewed in [43]), including its role in synaptic plasticity [10] and on specific amino acid deficiency [14], they have not explicitly addressed its impact on remyelination. Much evidence has supported ISR activation as a protective mechanism for oligodendrocytes during remyelination in mouse models of MS [27,29,30]. However, the role of GCN2 as a kinase driving the phosphorylation of eIF2α has not been defined so far. Here, by analyzing the mice deficient in the GCN2 sensor, we found that GCN2 is necessary for maintaining cortical myelin levels and proper myelin formation, as evidenced by reduced MBP segment length. We also discovered that under stress conditions, GCN2 participates in oligodendrocyte-mediated responses in a brain region-dependent manner. Specifically, while in the corpus callosum, GCN2 is necessary for effective remyelination; in the cortex, GCN2 is involved in both demyelination and remyelination and is required for eIF2α phosphorylation and eIF2α availability. 

Mature oligodendrocytes are responsible for myelination in the CNS. The role of GCN2 in oligodendrocyte biology, myelin formation, myelin removal, or MBP expression has not been previously described. Under normal conditions, the GCN2-deficient mice exhibited decreased levels of MBP and shorter myelin segments in the cortex, indicating that GCN2 participates in regulating MBP abundance and configuration. However, no observable difference in the degree of myelination was noted in the corpus callosum when compared to WT mice. These results suggest that the role of GCN2 we identified in myelination fine-tuning is also dependent on the brain region. 

MBP plays a crucial role in the formation and maintenance of the myelin sheath in the CNS, which is essential for efficient nerve impulse conduction and is also a major component of the myelin sheath, accounting for about 30% of its protein content [44]. MBP also participates in transmitting extracellular signals to the cytoskeleton in oligodendrocytes, and a signaling function has been proposed [45]. Its essential role is revealed since its absence or dysfunction can lead to a severely altered neurological phenotype [46]. 

In adults, an oligogenic source exists in the corpus callosum, where adult oligodendrocyte precursor cells develop into myelinating oligodendrocytes; this occurs to a lesser extent in the cortical gray matter [47]. During adulthood, NG2-positive cells, which are oligodendrocyte precursors, continue to generate oligodendrocytes in a brain region-specific manner [48]. The overall rate of oligogenesis in the cortex is significantly lower than that in the corpus callosum at most ages [49]. Our findings indicate that GCN2 deficiency particularly affects myelination in brain regions with lower oligogenic potential. This relationship between GCN2 and oligogenic potential may partially explain the variation in basal myelination status between the corpus callosum and cortex in GCN2-deficient mice. 

In the cortex, basal myelination in mice lacking GCN2 remains significant, but the decrease in MBP levels highlights the kinase’s role in regulating the abundance of essential myelin components under basal conditions. Conversely, the reduction in the average length of myelinated segments in the cortex of GCN2-deficient mice, as compared to wild-type mice, suggests that GCN2 is involved in fine-tuning the myelin segments.

It is still unclear how GCN2 participates in MBP level regulation. In mice, the genes encoding myelin components such as MBP, proteolipid protein (PLP) 1, PLP12, or myelin-associated protein (MAG) do not contain 5′ upstream open reading frames (uORFs) that can be controlled by eIF2α phosphorylation. This suggests that the regulation exerted by ISR activation, which impacts myelination, does not involve the translational control of the MBP transcript or other myelin-related transcripts. It indicates that GCN2 regulates the maturation and compaction processes in oligodendrocytes. It is intriguing that even when 37.3% of myelin is lost due to GCN2 deficiency, the mice still perform adequately under non-stress conditions. The precise amount of MBP necessary for normal CNS function in mice has not been clearly established. However, in a mouse that is highly deficient in MBP (>90%) due to a mutation in the MBP coding gene (shiverer mice), restoring 25% of MBP levels can rescue the severe and lethal phenotype exhibited by shiverer mice. The MBP levels in GCN2^−/−^ mice are sufficient for their normal performance. *Olig2* is a transcription factor essential for the specification, differentiation, and maturation oligodendrocyte precursor cells (OPCs) into myelin-producing cells [50]. In adults, the protein Olig2 helps maintain a population of OPCs capable of differentiating into mature oligodendrocytes [51] when needed, for example, in response to injury or normal myelin turnover [52]. However, if this increased Olig2 expression persists, as we found in GCN2^−/−^ mice, it can result in premature depletion of OPCs, eventually leading to a net decrease in MBP as fewer new oligodendrocytes are available for myelin production.

GCN2 triggers an adaptive response during amino acid deficiency in the brain [11,12,13]. The capacity for GCN2-mediated adaptation includes the translation of mRNAs with uORFs and gene reprogramming induced by eIF2α phosphorylation. Genes activated by GCN2 encompass those encoding proteins involved in autophagy, maintaining redox balance and amino acid metabolism. When challenged to demyelinate, GCN2^−/−^ mice, unlike WT mice, show severe sensitivity to cuprizone, with a significant proportion of the group dying during the challenge. Previous studies indicate that cuprizone treatment for eight weeks leads to amino acid deprivation, particularly impacting plasma levels of non-essential amino acids such as alanine, glycine, and proline [53]. Thus, the sensitivity phenotype observed in GCN2^−/−^ mice may arise from their inability to trigger an adaptive response during demyelination and subsequently facilitate remyelination, as WT mice do, possibly due to shifts in amino acid levels acting as a stimulus for GCN2 activation. Furthermore, our results showing an increase in eIF2α phosphorylation in WT mice during demyelination/remyelination, which is not detected in the GCN2^−/−^ mice (Figure 3A,B). We do not discard the possible contribution of other ISR kinases, like PERK and PKR, that also phosphorylate eIF2α under stress conditions. However, the low levels of phosphorylated eIF2α detected during remyelination in GCN2^−/−^ mice indicate that the adaptive capacity to manage the demyelinating stimulus and remyelination is highly mediated by the phosphorylation of eIF2α induced by GCN2. Since oligodendrocytes are the primary responders to demyelination-related stress, playing a crucial role in maintaining myelin integrity [30,54,55], part of the phenotype observed here could be due to the contribution of GCN2 in oligodendrocytes, but we cannot discard the contribution of another cell type. In MS mouse models, inducing this eIF2α phosphorylation-mediated adaptive capacity, either through genetic or pharmacological strategies, yields beneficial and protective effects in reducing EAE disease progression [27,29,30], plausibly driven by GCN2 activation. Notably, our results revealed that eIF2α levels are not recovered after the cuprizone challenge, suggesting that GCN2-deficient mice not only fail to trigger the adaptive response but may also lack any compensatory mechanisms driven by other eIF2α kinases as these mechanisms cannot be executed without the necessary substrate. Our data support a GCN2-mediated regulation of eIF2α expression. So far, the regulation of eIF2α expression remains poorly studied and limited to in vitro studies. It has been shown that during T-cell activation, eIF2α mRNA levels increase severalfold, with a subsequent rise in protein levels [56]. In this study, the authors proposed intranuclear eIF2α mRNA stabilization of the primary transcripts as the possible mechanism for increasing mRNA levels. Nuclear mRNA modifications, particularly mRNA methylation, regulate the stability of mRNAs, among other properties, like splicing and transport, which is critical for CNS myelination [57]. It remains to be explored whether modification on the eIF2α encoding transcript that occurs during remyelination affects its stability and depends on eIF2α kinases. At least under demyelination/remyelination stimuli, GCN2 seems to play a major role in controlling eIF2α. Moreover, our results suggest that eIF2α protein recovery could be part of the adaptive program induced by the activation of the ISR and, in the context of remyelination, triggered by GCN2. 

Here, we employed the cuprizone model to examine the role of GCN2 in remyelination, focusing on the signaling pathway that, based on our results, plays an important role in the process. Future research should explore GCN2 function in models that better capture the inflammatory components of MS. For instance, the lysolecithin-induced demyelination model could help assess how GCN2 influences focal lesion repair in an inflammatory microenvironment. Additionally, methionine-deficient models could provide critical insights into the metabolic regulation of the ISR in oligodendrocytes, further clarifying how amino acid availability affects eIF2α phosphorylation and myelin repair dynamics. These future studies would expand our understanding of GCN2’s broader relevance in demyelinating diseases and its potential as a therapeutic target.

Limitations of our study include the small sample size, which restricted the statistical power and generalizability of our findings. This constraint also hindered our ability to explore potential sex-based differences in the outcomes. The limited statistical power due to the small number of animals prevented us from drawing conclusions about how sex may influence the results. We recognize these limitations and suggest that future studies with larger sample sizes must address these issues and provide more insights into the effects of sex and other variables on the observed outcomes.

In summary, we demonstrated that GCN2 plays an essential role in oligodendrocyte function and normal remyelination of the CNS. We also showed that GCN2 contributes to the activation of the ISR during remyelination processes. Our findings support the idea of GCN2 as a potential target for demyelinating diseases beyond its immune function as it directly influences myelination and the adaptive response mediated by eIF2α. Future directions could focus on defining how GCN2 regulates myelin components and eIF2α expression.

## 4. Materials and Methods

### 4.1. Mice

Mice lacking the Eif2ak4 gene, which encodes the GCN2 protein (GCN2^−/−^), were obtained from Jackson Laboratories (Bar Harvor, ME, USA) (stock number 008240, B6.129S6-Eif2ak4tm1.2Dron/J). All animal care and experimental procedures adhered to the guidelines outlined in the “Guide for the Care and Use of Laboratory Animals” (Commission on Life Sciences, National Research Council, National Academy Press, Washington, DC, USA. 1996) and received approval from the Bioethical Committee of Fundación Ciencia and Vida (Protocol Number P029/2021). 

### 4.2. Cuprizone Intoxication Model

The study utilized 8-week-old male C57BL/6 mice, consisting of 17 WT and 29 GCN2-deficient individuals. Over a five-week period, the mice were fed a regular chow diet mixed with 0.2% (*w*/*w*) cuprizone (Sigma, St. Louis, MO, USA, C9012-25G) [32]. After the treatment, the mice were allowed to undergo remyelination for one week after the withdrawal of the drug, at which point they were euthanized. To ensure accurate comparisons, all biochemical and histological analyses were conducted on groups of littermates from the same breeding generation. To evaluate the general health of the mice during cuprizone treatment, kyphosis, eye closure, and hirsute fur were monitored before the treatment, once a week during the five weeks of cuprizone treatment, and once during the week of drug withdrawal. We assigned scores from 1 to 3 for minor, mild, and severe alteration, respectively. 

### 4.3. Tissue Processing for Immunofluorescence

Two groups of male WT (n = 3) or GCN2^−/−^ (n = 3) mice were used for the immunofluorescence assay. For this, mice were transcardially perfused with 0.9% NaCl until complete exsanguination. Subsequently, fixation was performed by transcardial perfusion with 50 mL of 4% PFA (in PBS1X) per mouse. The brains then were extracted and post-fixed in 4% PFA at 4 °C overnight. After fixation, the brains were washed three times in PBS1X for 10 min each at room temperature. Next, the samples were dehydrated by sequential immersion in 15% and 30% sucrose at 4 °C for 24 h each. Finally, the brain tissue was embedded in commercial media (OCT). Coronal brain sections (30 µm thick) were obtained using a microtome (LEICA, Wetzlar, Germany, CM1860UV) and mounted on glass slides. These slides were washed in PBS1X for 15 min at room temperature, then immersed in a solution containing citrate buffer (10 µM citric acid, 0.05% Triton X-100, pH 6) and microwaved for one minute until boiling to facilitate epitope exposure. Immediately after, the slides were agitated at room temperature until boiling ceased. The samples were then washed three times with PBS1X for 5 min each. Next, the samples were blocked with 1% horse serum in PBS1X for 1 h at room temperature. This was followed by overnight incubation at 4 °C with a monoclonal primary antibody against MBP (1:350, R&D Systems, Minneapolis, MN, USA, MAB42282). The following day, the samples were washed three times in PBS1X for 10 min each at room temperature. They were then incubated with an anti-mouse secondary antibody (Alexa Fluor 594 AffiniPure Donkey Anti-Mouse IgG (H + L), Jackson ImmunoResearch, West Grove, PA, USA, Cat. 715-585-150) (1:10,000), at 4 °C for 2 h. Finally, the samples were washed three times with PBS1X for 10 min each and mounted using a mounting medium (DAKO, Agilent, Santa Clara, CA, USA, S302380-2). The mounted samples were stored at 4 °C.

### 4.4. Determination of MBP-Positive Myelin Fragments

Three single-plane images of the motor cortex were captured per animal using an Olympus FV1200 confocal microscope at 40× magnification. Motor cortex images of equal total area were processed through intensity thresholding to isolate MBP-positive signals. These signals were then segmented via binarization in each image. The binary MBP-positive segments were quantified using the “Fit Elipse” macro from Fiji. Next, the pool of binary MBP-positive segments was refined by applying an area-size exclusion criterion based on objective resolution and reference standards. Segments with all circularities were included. Finally, MBP-positive segments were counted, and their lengths were measured in each image. Image denoising, segmentation, and quantification (segment counting and length determination) were carried out using Fiji software (version 2.9.0). The total number of segments per length of each strain was plotted. The mean myelin segment length for WT and GCN2^−/−^ motor cortices was determined from the total segment pool of each strain. A diagram is shown in Supplementary Figure S1A,B.

### 4.5. Tissue Processing for Immunohistochemistry

The animals were perfused with 50 mL of phosphate-buffer saline. Brains were removed and halved coronal. One hemisphere (anterior) was fixed with 4% paraformaldehyde in PBS and processed for immunohistochemical analysis. The posterior region was used for biochemical analysis and stored at −80 °C. After the week on PFA 4%, the samples were dehydrated by an alcohol battery (from 50, 70, 80, 90 until 100%), then were paraffin-embedded, and 12 μm coronal sections were cut in a microtome (Microm HM 325, Thermo Scientific, Waltham, MA, USA). Sections were re-hydrated through an alcohol battery (100, 90, 80, 70, 60 until 50%), and epitopes were exposed to citrate buffer pH 6 (30 min at 65 °C). Then, endogenous peroxidase removal through hydrogen peroxide 3% treatment for 30 min in agitation. Then, samples were blocked with a blocking solution (Vector Laboratories, Newark, CA, USA, PK-6102) for 1 h. MBP immune detection was assessed by primary antibody anti-MBP (Aves Lab, Davis, CA, SUA, 1:1000) dissolved in TBS 1X (Tris-HCL 100 mM, NaCl 0.9%) and the Kit Anti-Mouse IgG (Vector Laboratories, Newark, CA, USA PK-6102). The images were captured by epifluorescence microscopy (Olympus, Tokyo, Japan, BX51). 

### 4.6. Western Blot

Tissues were collected and stored at −80 until preparation. For tissue analysis, the tissue was homogenized and sonicated in buffer PBS 1X containing phosphatase and protease inhibitor cocktail 1X. The protein concentration was determined by BCA assay (Pierce Thermo Fisher Scientific, Waltham, MA, USA, 23225). The amount of protein loaded is indicated in each figure. For MBP detection, anti-MBP (CAT# MAB42282, R&D Systems, Minneapolis, MN, USA) (1:1000) dissolved in buffer PBS 1X was used. For eIF2α, detection was performed with the Cell Signaling (Danvers, MA, USA) #9722S (1:1000) antibody dissolved in TBS 1X buffer (with no fat dry milk 5%). For phosphorylated eIF2α, (Cell Signaling, Danvers, MA, USA) #3398S (1:1000) antibody was dissolved in TBS 1X (with BSA 5%), using this as a secondary antibody anti-rabbit IgG cross-absorbed HRP (Invitrogen, Carlsbad, CA, USA, #31462). For α-Tubulin detection (Invitrogen, Carlsbad, CA, USA) #32-2500 (1:5000) antibody dissolved in PBS 1X (with no fat dry milk 5%) was used. The membrane was revealed with the Chemidoc Touch system (Chemidoc Imaging System, Bio-RAD, Hercules, CA, USA, software version 2.4.0.03). The images were analyzed with ImageJ software (1.53t version).

### 4.7. Quantitative PCR

mRNA was extracted from samples through the conventional Trizol protocol (Invitrogen, Carlsbad, CA, USA, Cat. 10296010). An amount of 1.5 µg/µL was used to synthesize cDNA. cDNA was synthesized using the kit Thermo (Waltham, MA, USA) #4368813, obtaining 2 μg/μL cDNA. Sybr Green (Agilent, Santa Clara, CA, USA) #600828 protocol was performed using the following primers: against eIF2α forward: 5′-AGT TGT AGG TTA GGC GTC CC-3′ and reverse:5′-ACT ACT GCA CTC CTT CGA CC-3′, actin forward: 5′-TAC CAC CAT GTA CCC AGG CA-3′ and reverse: 5′-CTC AGG AGG AGC AAT GAT CTT GAT-3′, Olig2 forward: 5′-ATC CCG GGG ACA AAC TGG-3′ and reverse: 5′-TG TTG ATC TTC AGG CGC AG-3′, CNP forward 5′-CCT GGA GAA GTA CCA CGA CG-3′ and reverse: 5′ -GTC TAG ACG CTT GTA CGC CT-3′.

## 5. Conclusions

Our findings highlight the importance of GCN2 in regulating the levels of myelin components necessary for both myelination and the refinement of myelin in the CNS. We also found that GCN2 is crucial for remyelination following demyelination. In addition, we demonstrate the role of the nutrient sensor in phosphorylating eIF2α under normal conditions, as well as during demyelinating and remyelination and in regulating eIF2α expression under stress conditions in the CNS.

## Data Availability

The datasets used and/or analyzed during this current study are available from the corresponding author on reasonable request.

## Supplementary Materials

The following supporting information can be downloaded at: https://www.mdpi.com/article/10.3390/ijms26041626/s1.
