# Supplementary material for: GCN2-Mediated eIF2α Phosphorylation Is Required for Central Nervous System Remyelination
Source: Int J Mol Sci. 2025 Feb 14;26(4):1626. doi: 10.3390/ijms26041626 (PMC11855834; doi:10.3390/ijms26041626)
Supplement: Supplementary file 1 [file ijms-26-01626-s001.zip › ijms-3411023-supplementary.pdf]

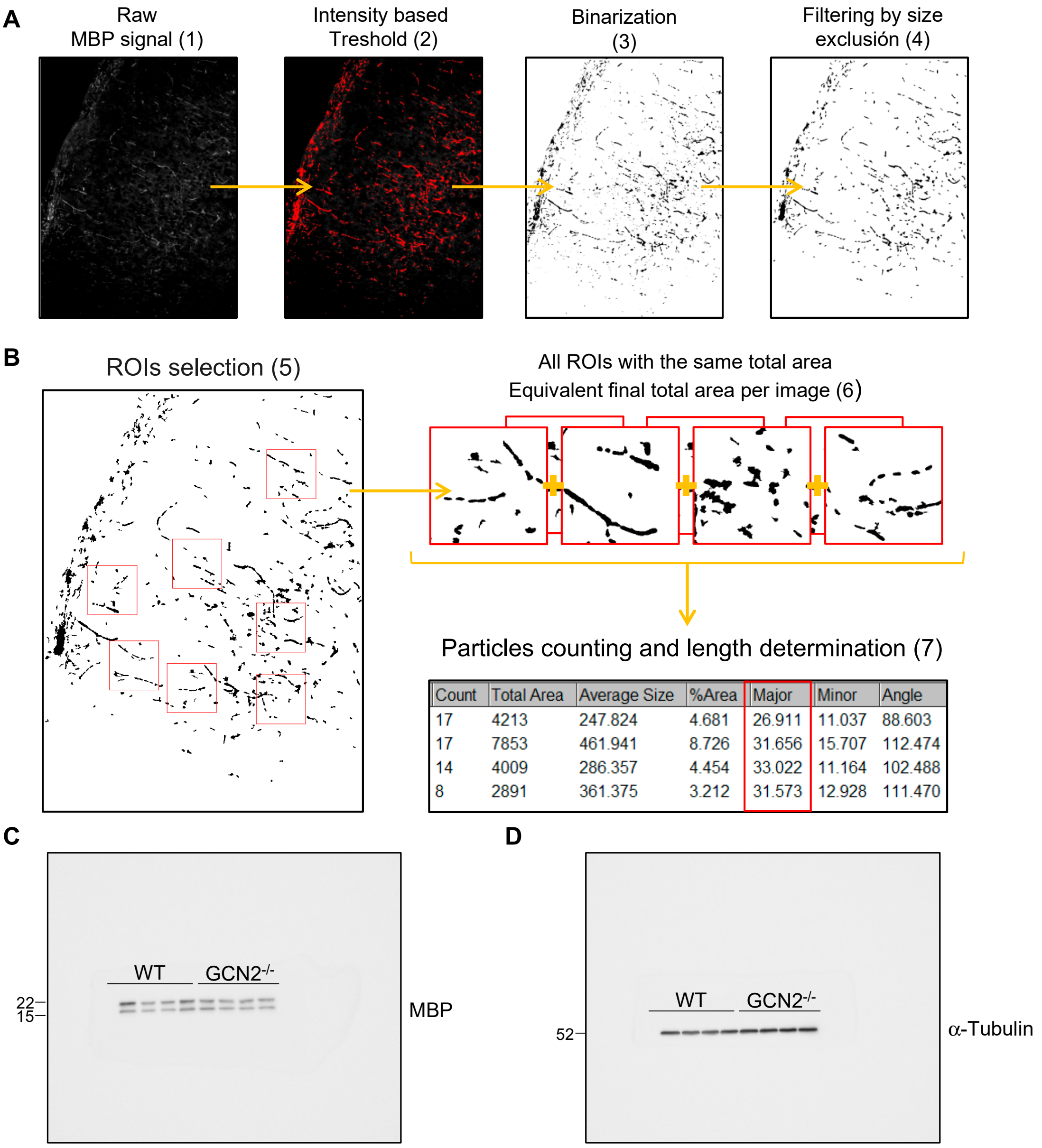

Supplementary Figure S1. Image processing for MBP segments analysis and whole membranes of WT and GCN2$^{-/-}$ mice. Single-plane motor cortex images from MBP-stained immunofluorescences were obtained using an OLYMPUS FV1200 confocal microscope at 40X magnification. **A**) Raw MBP signal (1) was thresholded based on intensity (2) to isolate MBP-positive areas from background intensities. Afterward, areas above the intensity threshold were binarized (3). Then, binarized particles were filtered by an area-size exclusion criterion based on objective resolution and reference standards (4). **B**) Regions of interest (ROIs) were selected to include representative particle populations in each image (5). The total area selected was the same for each image (6). Finally, MBP-positive segments were counted, and their length was determined (7). The whole membrane corresponds to the Western blot shown in Figure 1C, for **C**) WT mice and **D**) GCN2$^{-/-}$ mice. The membrane was cut at 38 kDa and simultaneously incubated with anti-MBP and anti-α-Tubulin antibodies.

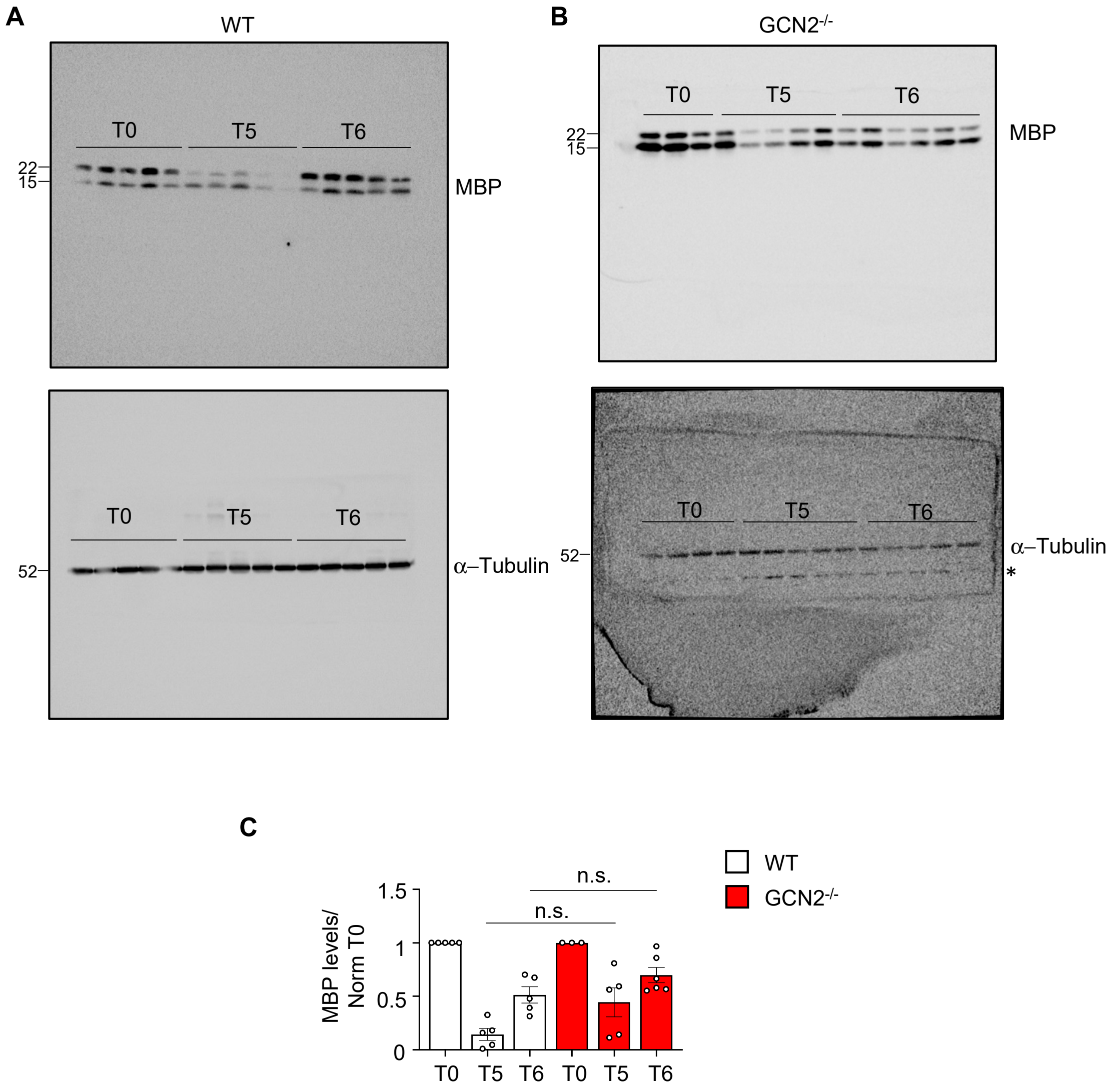

Supplementary Figure S2. The membranes correspond to the Western blot shown in Figure 2C and 2D. **A**) Analysis of WT mice and **B**) GCN2 deficient mice (GCN2$^{-/-}$). Detection of MBP and α-Tubulin was performed in the same membrane for each genotype. *, indicates a nonspecific band. **C**) Analysis from data presented in Figure 2C and 2D. The MBP levels found after five weeks of cuprizone treatment (T5) and after one week of recovery (T6) from WT (white bars) and GCN2$^{-/-}$ (red bars) mice were normalized against values obtained on basal conditions (T0). and then statistical analyses were conducted accordingly. Mann Whitney n.s.: non-significative differences. Error bars represent the mean ± SEM.

WT (N=6)	T0	T5	T6
Eye opening	0	1	1
Hirsute hair	0	3	1
Kyphosis	0	1	1

GCN2$^{-/-}$ (N=12)	T0	T5	T6
Eye opening	0	1	1
Hirsute hair	0	3	3
Kyphosis	0	3	3

Supplementary Figure S3. We monitored the general health of the mice before (T0), during five weeks of treatment (T5), and after 1 week of cuprizone treatment (T6). We assessed kyphosis (spinal curvature), eye closure, and hirsute fur, and based on the mice's condition, we assigned scores from 1 to 3 for minor, mild, and severe alterations, respectively. The average scores for WT (N=8) and GCN2-/- (N=18) are shown for each parameter.

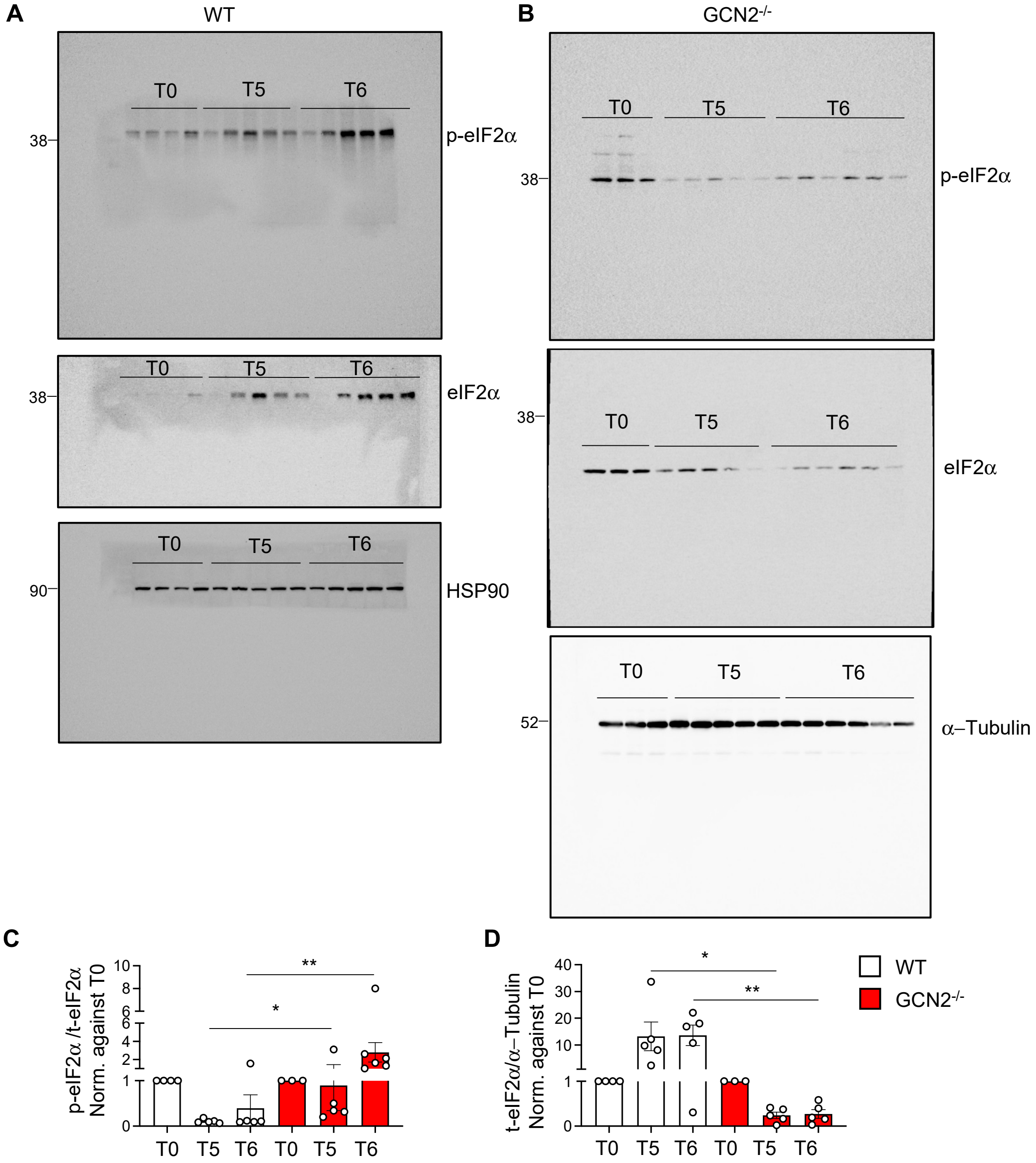

Supplementary Figure S4. The membranes correspond to the Western blots shown in Figures 3A and 3B. **A**) Analysis of WT mice and **B**) GCN2 deficient mice (GCN2$^{-/-}$). Detection of total eIF2α, p-eIF2α, and loading control were performed in the same membrane for each genotype. For both membranes, we incubated the p- eIF2α antibody; then the membrane was stripped, and we incubated them with the eIF2α antibody. Finally, we blotted against α-Tubulin or HSP90. **C**) Analysis from data presented in Figure 3A for phosphorylated eIF2α levels (p-eIF2a) levels and **D**) for total eIF2α levels (eIF2a), normalized against α-Tubulin. The p-eIF2a levels or eIF2a levels found after five weeks of cuprizone treatment (T5) and after one week of recovery (T6) from WT (white bars) and GCN2$^{-/-}$ (red bars) mice were normalized against values obtained on basal conditions (T0). Mann Whitney *$p <$ 0,05; **$p <$ 0,01; Error bars represent the mean ± SEM..

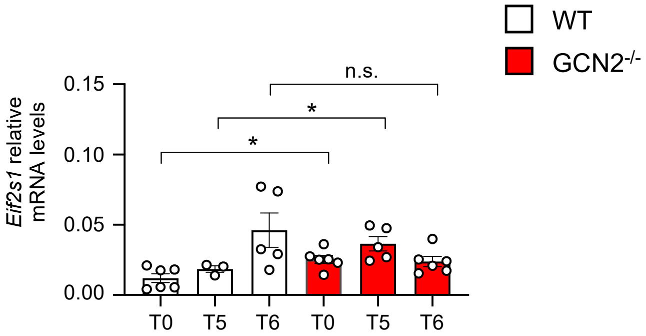

Supplementary Figure S5. WT and GCN2$^{-/-}$ mice were treated with cuprizone, and the levels of *Eif2s1* transcript, encoding for eIF2α, were evaluated on total mRNA obtained from the cerebral cortex of WT mice untreated (T0, N=6), after 5 weeks of treatment with cuprizone (T5, N=3), and at the sixth week of regular chow after 5 weeks of treatment (T6, N=5) and from GCN2$^{-/-}$ mice untreated (T0, N=6), after 5 weeks of treatment with cuprizone (T5, N=5), and at the sixth week of regular chow after 5 weeks of treatment (T6, N=6). The plot shows *Eif2s1* levels normalized against *Actin* transcript levels and the statistical significance between genotypes at T0, T5 and T6. Mann Whitney test. n.s.: non-significative differences. **p*<0,05. Error bars represent the mean ± SEM.

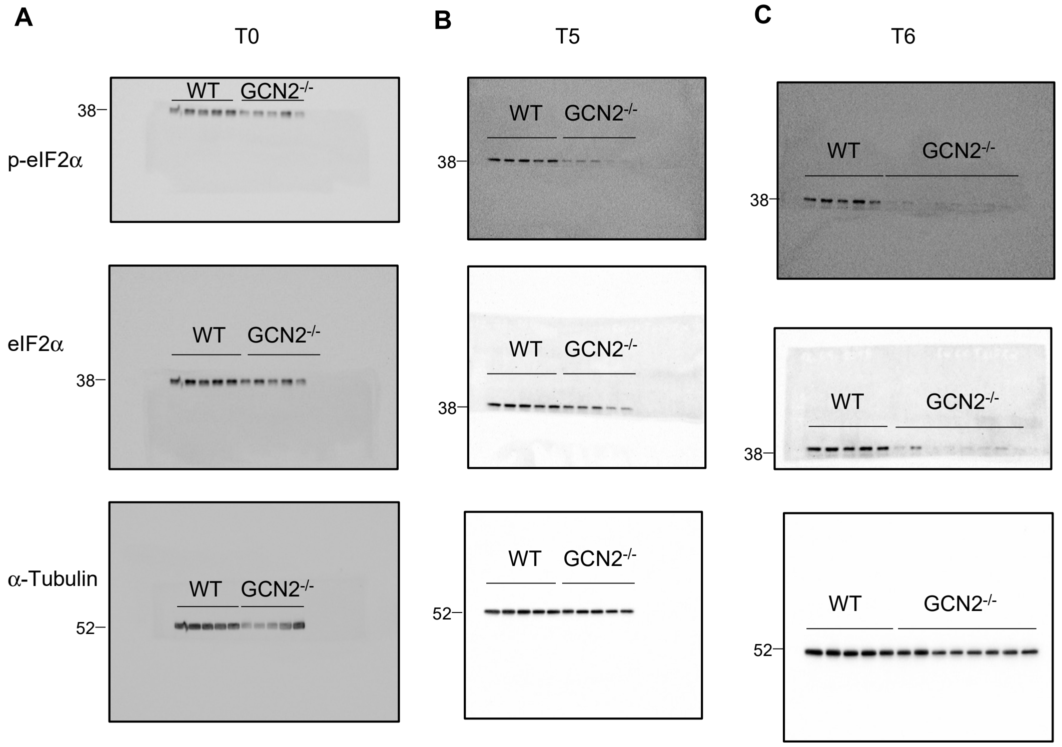

Supplementary Figure S6. The membranes correspond to the Western blots shown in Figure 4. The levels of the α subunit of the eukaryotic translation initiation factor 2 (eIF2α) and its phosphorylated form (p-eIF2α) between WT and GCN2 deficient mice (GCN2$^{-/-}$) were analyzed using Western blot at **A**) basal conditions (T0), **B**) after treatment with cuprizone for five weeks (T5) and **C**) at the sixth week of regular chow after 5 weeks of treatment (T6). For all membranes, we incubated the p-eIF2α antibody, and after the membrane was stripped, we incubated it with the eIF2α antibody. Finally, we blotted them against α-Tubulin as the loading control.

A

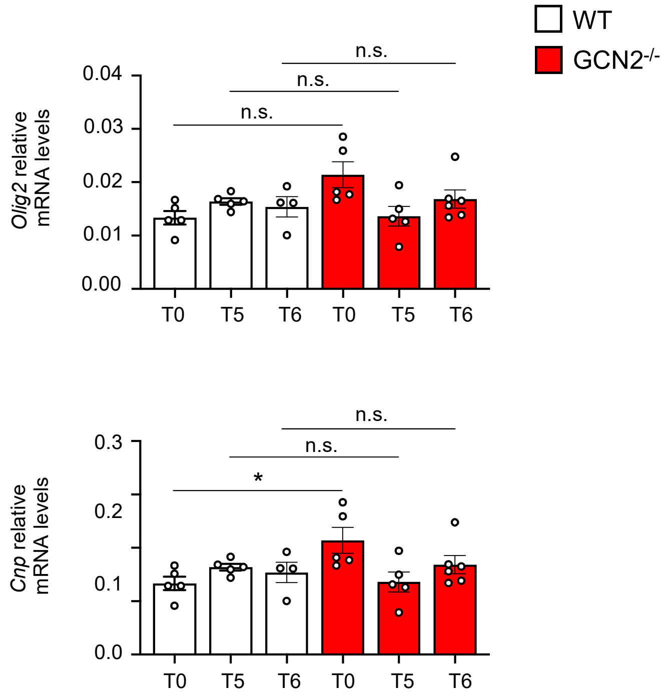

Supplementary Figure S7 WT and GCN2$^{-/-}$ mice were treated with cuprizone, and the levels of *Eif2s1* transcript, encoding for eIF2α, were evaluated on total mRNA obtained from the cerebral cortex of WT mice untreated (T0, N=6), after 5 weeks of treatment with cuprizone (T5, N=3), and at the sixth week of regular chow after 5 weeks of treatment (T6, N=5) and from GCN2$^{-/-}$ mice untreated (T0, N=6), after 5 weeks of treatment with cuprizone (T5, N=5), and at the sixth week of regular chow after 5 weeks of treatment (T6, N=6). The plot shows *Eif2s1* levels normalized against *Actin* transcript levels, and the statistical significance between the time points is shown. t-test. n.s.: non-significative differences. **p*<0,05. Error bars represent the mean ± SEM.
